# VEGF-B promotes recovery of corneal innervations and trophic functions in diabetic mice

**DOI:** 10.1038/srep40582

**Published:** 2017-01-16

**Authors:** Guohu Di, Xiaowen Zhao, Xia Qi, Songmei Zhang, Lu Feng, Weiyun Shi, Qingjun Zhou

**Affiliations:** 1 State Key Laboratory Cultivation Base, Shandong Provincial Key Laboratory of Ophthalmology, Shandong Eye Institute, Shandong Academy of Medical Sciences, Qingdao, China

## Abstract

Vascular endothelial growth factor (VEGF)-B possesses the capacity of promoting injured peripheral nerve regeneration and restore their sensory and trophic functions. However, the contribution and mechanism of VEGF-B in diabetic peripheral neuropathy remains unclear. In the present study, we investigated the expression and role of VEGF-B in diabetic corneal neuropathy by using type 1 diabetic mice and cultured trigeminal ganglion (TG) neurons. Hyperglycemia attenuated the endogenous expression of VEGF-B in regenerated diabetic corneal epithelium, but not that of VEGF receptors in diabetic TG neurons and axons. Exogenous VEGF-B promoted diabetic corneal nerve fiber regeneration through the reactivation of PI-3K/Akt-GSK3β-mTOR signaling and the attenuation of neuronal mitochondria dysfunction via the VEGF receptor-1 and neuropilin-1. Moreover, VEGF-B improved corneal sensation and epithelial regeneration in both normal and diabetic mice, accompanied with the elevated corneal content of pigment epithelial-derived factor (PEDF). PEDF blockade partially abolished trophic function of VEGF-B in diabetic corneal re-innervation. In conclusion, hyperglycemia suppressed endogenous VEGF-B expression in regenerated corneal epithelium of diabetic mice, while exogenous VEGF-B promoted recovery of corneal innervations and trophic functions through reactivating PI-3K/Akt-GSK-3β-mTOR signaling, attenuating neuronal oxidative stress and elevating PEDF expression.

Diabetic peripheral neuropathy (DPN) is one of the most common and irreversible complications in both type 1 and type 2 diabetes mellitus, among which the earliest alterations occur in the small unmyelinated C- and thinly myelinated Aδ-nerve fibers^1^,^2^. As the most densely innervated tissue in the body, the corneal nerve fibers are highly affected by hyperglycemia, such as decreased nerve fiber density, reduced length and increased tortuosity^3^,^4^,^5^. Recently, the quantification of corneal nerve fiber changes by using corneal confocal microscopy has been used for the rapid diagnosis and stratification of diabetic peripheral neuropathy^6^,^7^,^8^. Moreover, the attenuation of corneal sensory and trophic functions by hyperglycemia always predisposes diabetic patients to the symptoms of impaired corneal sensation and delayed epithelial wound healing, known as diabetic keratopathy clinically^9^. Previous studies have reported the roles of various neuropeptides and neurotrophic factors in the regulation of corneal epithelial wound healing and nerve fiber regeneration^10^,^11^,^12^,^13^,^14^. However, the mechanism of corneal neuropathy in diabetic mellitus remains unclear, and no effective treatment exists except the careful glycemic control for diabetic patients.

Vascular endothelial growth factor (VEGF) family has been shown to exert pleiotropic effects on both central and peripheral nervous system^15^,^16^. As the most well-known member of VEGF family, the translation of VEGF-A into clinical practice was severely compromised due to the concern of inducing undesired neovascularization in avascular cornea. In comparison with VEGF-A, VEGF-B has very restricted angiogenic activity, while also provides with neurotrophic and neuroprotective effects both *in vitro* and *in vivo*^17^,^18^. In cornea, *Vegf-b*^*−/−*^ mice exhibited no abnormal change of corneal nerve density and distribution, but assumed significant impaired corneal nerve fiber regeneration, sensation and epithelial wound healing. Whereas, exogenous VEGF-B application selectively induced the regeneration of injured corneal nerve fiber and corneal epithelial wound healing in both wide-type and *Vegf-b*^*−/−*^ mice^19^. Moreover, the promotion of VEGF-B on corneal epithelial wound healing was independent on the epithelial cell proliferation, which was likely due to the trophic functional recovery of regenerated corneal nerve fibers. However, the mechanism of VEGF-B on the recovery of injured corneal nerve trophic function and its contribution in the diabetic corneal neuropathy remains incompletely understood.

Previous and our studies have confirmed that streptozotocin injection-induced type 1 diabetic mice assumed typical characteristics of diabetic corneal neuropathy, including impaired corneal nerve regeneration with concomitant attenuated corneal sensory and trophic functions^10^,^20^,^21^. Given the capacity of VEGF-B to restore the anatomic and function corneal innervations in normal mice, here we explored the contribution and mechanism of VEGF-B in diabetic corneal nerve fiber regeneration, corneal sensation and trophic function recovery by using type 1 diabetic mice and cultured trigeminal ganglion neurons.

## Results

### Hyperglycemia causes impaired corneal innervations

**Figure 1:**
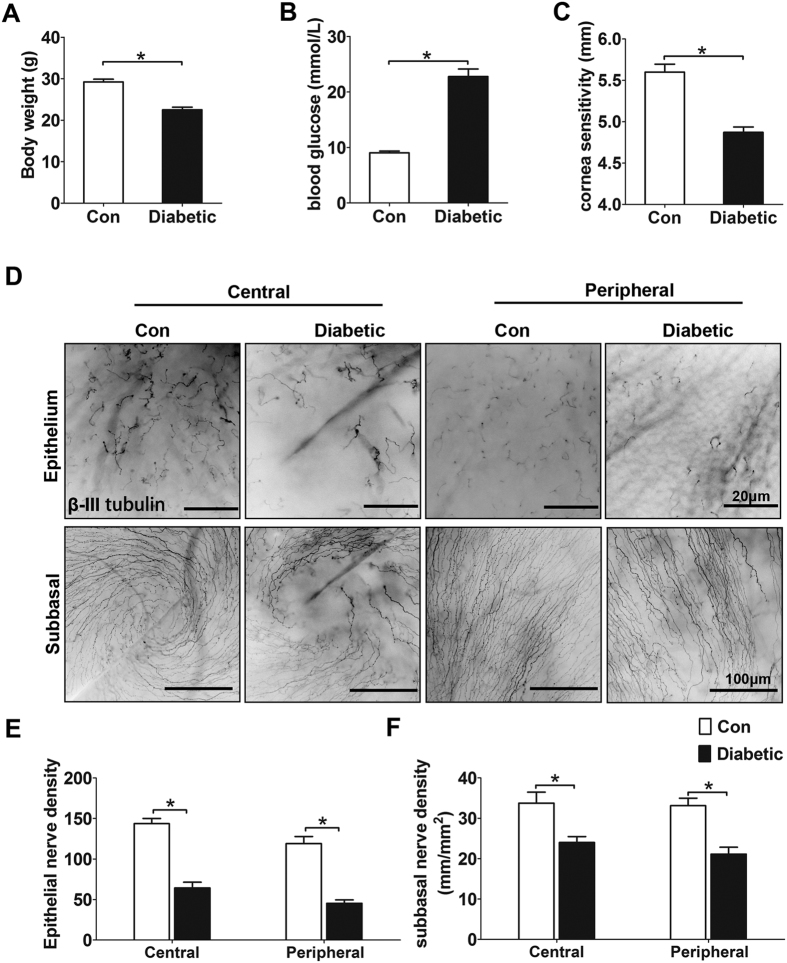
Index of STZ-induced diabetic mice over 4-month duration. (**A**) Body weight was reduced significantly in the STZ-treated mice compared with age matched control mice (n = 10 per group). (**B**) Blood glucose levels in the STZ-treated mice were significantly higher than that of control mice (n = 10 per group). (**C**) Corneal sensitivity was markedly decreased in the STZ-treated mice (n = 10 per group). (**D**) Representative images of neuronal β III tubulin-stained nerves of epithelium (top) and images of subbasal (bottom) in 4 month diabetic and age matched control corneas. The nerve density of terminal epithelium (**E**) and subbasal nerve plexus (**F**) were significantly decreased in STZ-treated mice at both central and peripheral cornea (n = 10 per group). Data are representative of means ± SD. *p < 0.05.

To investigate the impairment of corneal innervations by hyperglycemia, streptozotocin- induced type 1 diabetic mouse model was used according to our previous reports^10^,^13^. Body weight, blood glucose level, corneal sensitivity, corneal subbasal and epithelial nerve fiber density were monitored at 4 months after the final streptozotocin injection (n = 10 per group). The weight gain of diabetic mice was more restricted than that of age-matched normal mice (Fig. 1A), while their blood glucose was maintained over 25.0 mmol/L for 4 months (Fig. 1B). Accompanied with prolonged hyperglycemia duration, the mice exhibited a significant impairment in corneal sensitivity (Fig. 1C). Moreover, the whole-mount corneal staining showed that the density of subbasal nerve plexus and nerve ending in corneal epithelium was significantly decreased in diabetic mice than that of normal mice (Fig. 1D–F).

### Hyperglycemia suppresses VEGF-B expression in regenerated corneal epithelium, but not VEGF-B receptors in trigeminal ganglion neurons

**Figure 2:**
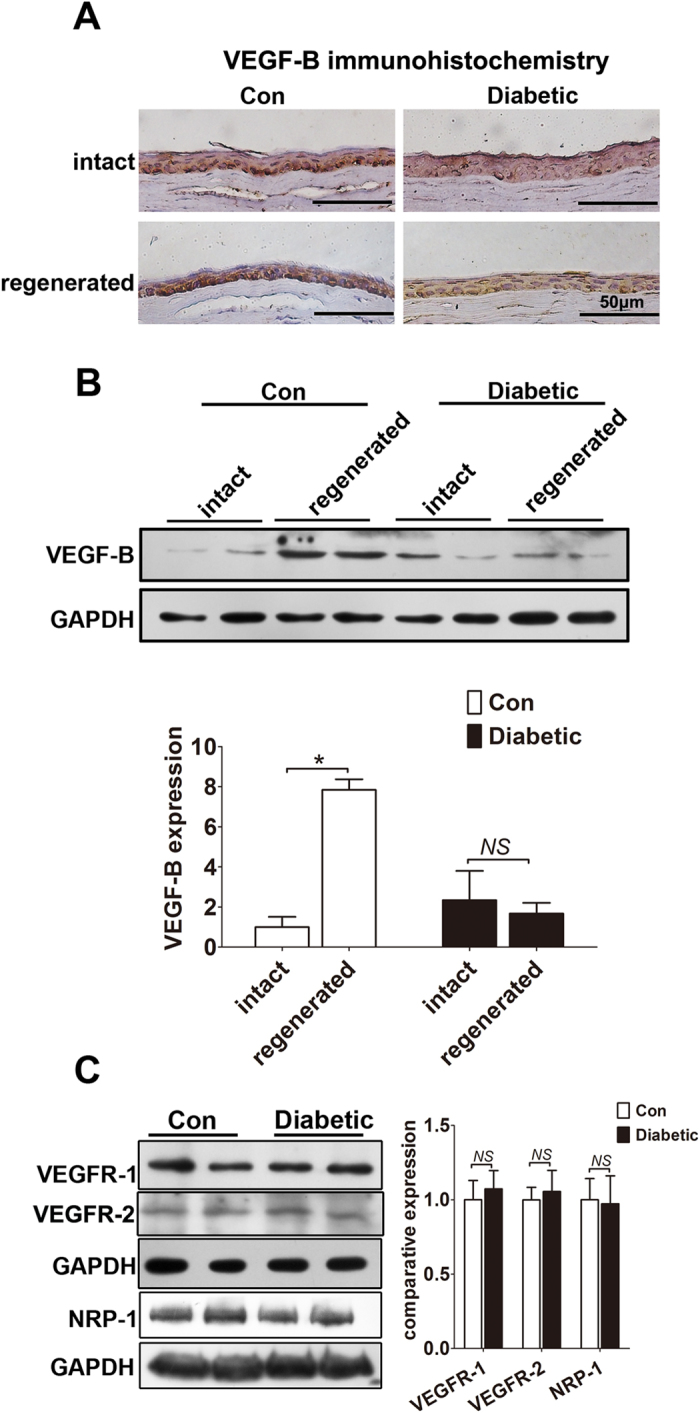
Hyperglycemia suppresses VEGF-B expression in regenerated corneal epithelium, but not their receptors in trigeminal ganglion neurons. (**A**) Diabetic mice and age matched control mice corneas were injured by 2mm central corneal epithelial debridement, 7 day after the scrape, the corneal samples was collected and stained with VEGF-B. (**B**) The regenerated corneal epithelium after 7 day the scrape was collected, 4 corneal epithelia were mixed as one sample and the expression of VEGF-B was analyzed by Western blot. (**C**) TG tissues from diabetic and normal mice were collected, expression of VEGFR-1, VEGFR-2 and NRP-1 were analyzed by Western blot. Data are representative of means ± SD. *p < 0.05.

Previous studies have showed that endogenous VEGF-B expression was increased in corneal epithelium and stroma after corneal nerve injury^19^. To investigate the possible influence of VEGF-B expression by hyperglycemia, the corneal epithelial debridement model was established in normal and diabetic mice, which accompanied with the injury and regeneration of corneal epithelium and nerve fibers. As shown in Fig. 2A, VEGF-B showed positive staining in the intact corneal epithelium of normal mice, while exhibited a significant enhanced staining in the regenerated corneal epithelium after 7 days of injury. According to the quantitative analysis of Western blot results, VEGF-B protein level was up-regulated by 7.8 folds in the regenerated corneal epithelium than that in intact epithelium of normal mice (Fig. 2B). Intriguingly, the expression of VEGF-B showed no obvious elevation in the regenerated diabetic corneal epithelium after injury, although there was also no significant difference in the intact corneal epithelium between normal and diabetic mice (Fig. 2A,B). In addition, the expressions of VEGF receptors, including VEGF receptor-1, VEGF receptor-2, and their co-receptor NRP-1, were broadly distributed over the trigeminal ganglion neurons and their axons (see Supplementary Fig. S2). However, they showed no statistical decreasing in the neurons of diabetic mice when compared with that of normal mice (Fig. 2C). In conclusion, hyperglycemia suppresses endogenous VEGF-B expression in regenerated corneal epithelium, but has no influence on VEGF-B receptor expressions in trigeminal sensory neurons.

### Exogenous VEGF-B promotes corneal innervations in diabetic mice

**Figure 3:**
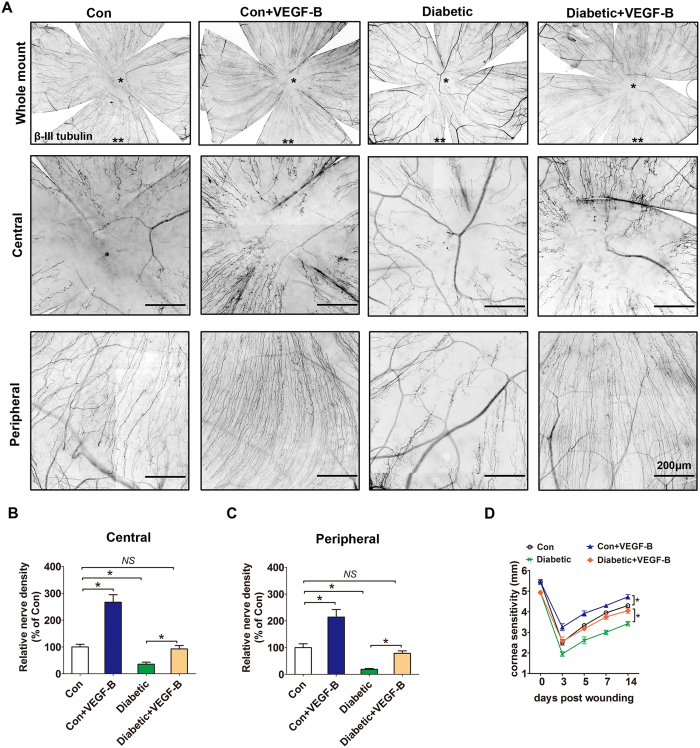
Exogenous VEGF-B promotes corneal innervations in diabetic mice. Diabetic and control mice were subjected to corneal epithelial debridement and subsequently injected subconjuntivally with 0.5 ng of recombinant mouse (rm) VEGF-B186 or an equal volume of PBS as control. (**A**) 7 days post wound, corneas were harvested, flat mounted, and stained with βIII-tubulin antibody. Representative images of subbasal nerve plexus in the central (*denoted) and peripheral cornea (**denoted) were taken. (**B**) Analysis of corneal subbasal nerve density in central cornea at 7 days post injury (n = 8 per group). (**C**) Analysis of corneal subbasal nerve density in peripheral cornea at 7 days post injury (n = 8 per group). (**D**) The corneal sensation was measured at appointed time post injury (n = 8 per group). Data are representative of means ± SD. *p < 0.05.

Hyperglycemia impairs corneal nerve fiber regeneration and re-innervations in the diabetic animals and human with the injured corneal epithelium^22^. To examine the effect of exogenous VEGF-B on diabetic corneal nerve regeneration, recombinant mouse VEGF-B186 was subconjunctivally injected immediately after the debridement of central corneal epithelium in normal and diabetic mice. The density of regenerated subbasal corneal nerve fibers was examined at 7 days after corneal epithelial debridement. As previous reports, VEGF-B promoted the subbasal nerve fiber regeneration in normal mice (Fig. 3A). Moreover, diabetic mice showed significantly delayed regeneration after epithelial injury, while VEGF-B supplement exhibited faster subbasal nerve fiber regeneration than vehicle PBS group, both at the central and peripheral areas of the cornea (Fig. 3B,C). In consistent with the nerve regeneration, VEGF-B significantly improved the corneal sensation recovery in diabetic mice (4.05 ± 0.24) and reached the equal level of normal mice (4.3 ± 0.18) after 14 days (Fig. 3D).

### VEGFR-1 mainly mediates the promotion of VEGF-B on neurite outgrowth

**Figure 4:**
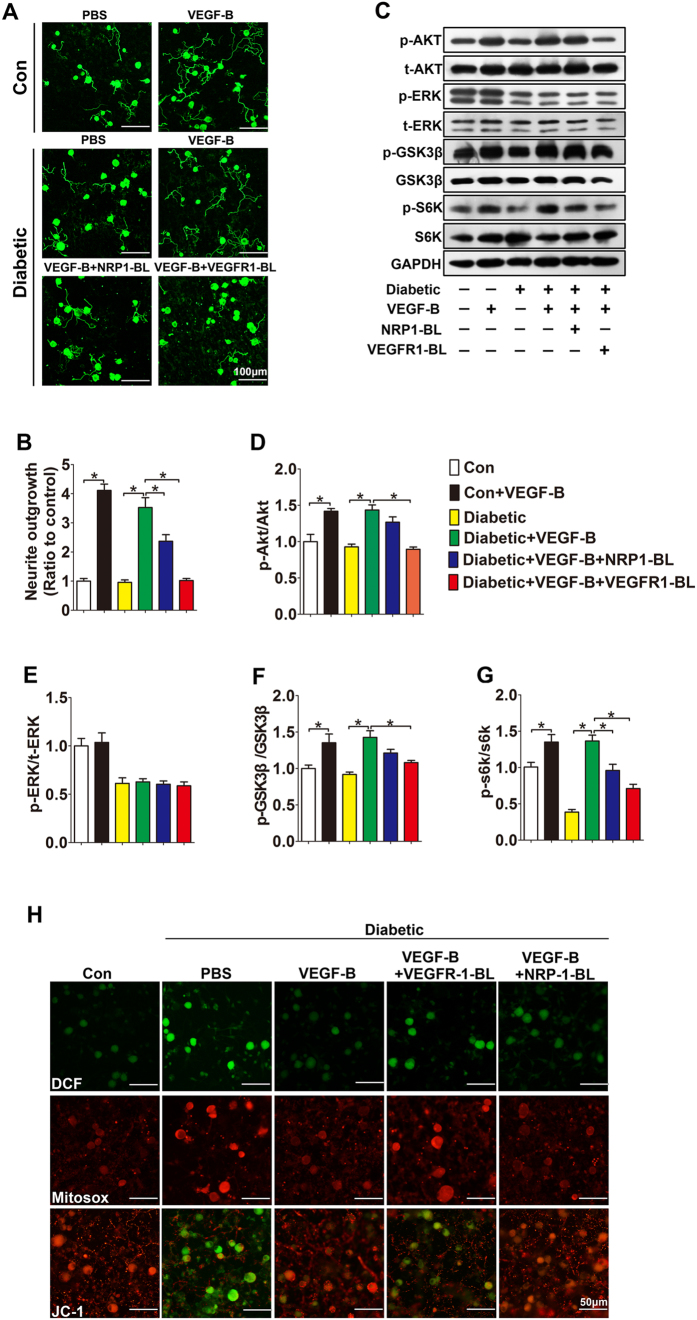
VEGFR-1 mainly mediates the promotion of VEGF-B on neurite outgrowth. Neurons from TG of diabetic or control mice were isolated and cultured as described in Material and Methods section. (**A**) Representative images of cultured TG neurons staining with βIII-tubulin antibody. (**B**) The total neurite length was analyzed with Neuron J software (n = 5 per group). (**C**) Cell lysate were collected, expression and phosphorylation state of Akt, ERK, GSK3β and s6k were analyzed by Western blot. (**D–G**) Quantification of Western blot results. (**H**) Representative images of TG neurons showing fluorescence of DCF, Mitosox and JC-1. Data are representative of means ± SD. *p < 0.05.

Corneal nerve fibers are mainly originated from the ophthalmic division of the trigeminal ganglia (TG). To explore the role of VEGF-B in diabetic nerve regeneration *in vitro*, TG neurons from diabetic mice were cultured in the medium with high glucose (25 mM) to recapitulate the hyperglycemia *in vivo*. As expected, after 48 hours, VEGF-B_186_ treatment significantly enhanced neurite elongation in TG neurons from both diabetic (3.5 folds) and normal mice (4.2 folds) (Fig. 4A,B). However, when the TG neurons were treated with the blocking antibodies specialized for VEGF-B receptors, the neutralization of VEGF receptor-1 (VEGFR-1) completely abolished, while neuropilin-1 (NRP-1) neutralization partially inhibited VEGF-B-stimulated neurite elongation in diabetic TG neurons. Moreover, treatment of diabetic TG neurons with VEGF-B significantly increased the levels of phosphorylated Akt, GSK3β and S6K (Fig. 4C–F). However, the phosphorylated level of ERK showed no significant improvement with VEGF-B treatment (Fig. 4C,G). Considering to the importance of oxidative stress in diabetic neuropathy, we further evaluated the regulations of VEGF-B on the intracellular reactive oxygen species (ROS) accumulation and mitochondrial dysfunction in cultured diabetic TG neurons. Hyperglycemia caused significant intracellular ROS (DCF staining) accumulation, mitochondrial superoxide generation (Mitosox staining), and the loss of mitochondrial membrane potential (red to green staining of JC-1). However, VEGF-B treatment attenuated ROS accumulation and mitochondrial superoxide generation, accompanied by the recovery of mitochondrial membrane potential (Fig. 4H, see Supplementary Fig. S3). Consistent with the inhibition of VEGF receptor blocking antibody on neurite elongation, VEGF-B failed to induce AKT, GSK3β, S6K activation and oxidative stress attenuation when pretreated with VEGFR-1 neutralizing antibody, while only partially impaired by NRP-1 neutralizing antibody (Fig. 4D–H). The results suggest VEGFR-1 mainly mediates the promotion of VEGF-B on the neurite outgrowth in diabetic TG neurons.

### VEGF-B reactivates neuronal PI-3K/Akt-GSK-3β-mTOR signaling altered by hyperglycemia

**Figure 5:**
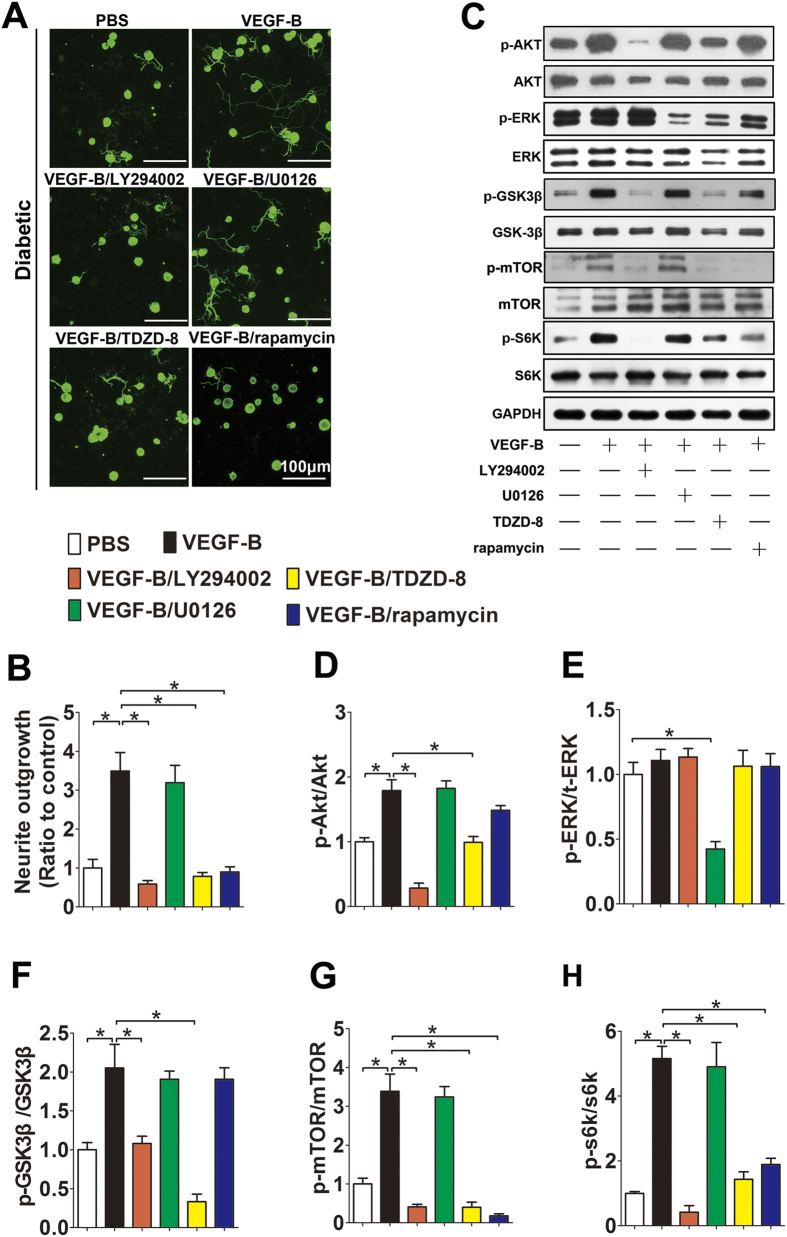
Intracellular signaling that mediated VEGF-B promotion on diabetic nerve fiber regeneration. Neurons from TG of diabetic mice were isolated and treated as described in Material and Methods section. (**A**) Representative images of TG neurons staining with βIII-tubulin antibody. (**B**) The total neurite length was analyzed with Neuron J software (n = 5 per group). (**C**) Expression and phosphorylation state of Akt, ERK, GSK3β, mTOR and s6k were analyzed by Western blot. (**D–H**) Quantification of Western blot results. Data are representative of means ± SD. *p < 0.05.

To delineate the critical signaling pathways activated by VEGF-B in diabetic sensory neurons, the TG neuron culture of diabetic mice were pretreated with pharmacological inhibitors in the presence of VEGF-B, including LY294002 for PI-3K/Akt inhibition, TDZD8 for GSK3β inhibition, rapamycin for mTOR inhibition, U0126 for ERK inhibition. The results showed that the inhibition of PI-3K/Akt, GSK3β or mTOR, but not the inhibition of ERK, completely abolished the enhanced neurite outgrowth of diabetic TG neurons induced by VEGF-B (Fig. 5A,B). Moreover, Western blot analysis revealed that VEGF-B activated mTOR and its downstream effector S6K, a molecule essential for protein synthesis and axonal growth, was entirely abolished by LY294002, TDZD8, or rapamycin, but not U0126. Meanwhile, VEGF-B activated GSK3β was inhibited by the PI-3k/Akt inhibitor, and followed by the blocking of S6K phosphrylation. Taken together, the results suggest PI-3K/AKT-GSK-3β-mTOR signaling mediates the promotion of VEGF-B on neurite outgrowth in diabetic TG neurons (Fig. 5C–H).

### VEGF-B accelerates diabetic corneal epithelial regeneration and modulates PEDF release

**Figure 6:**
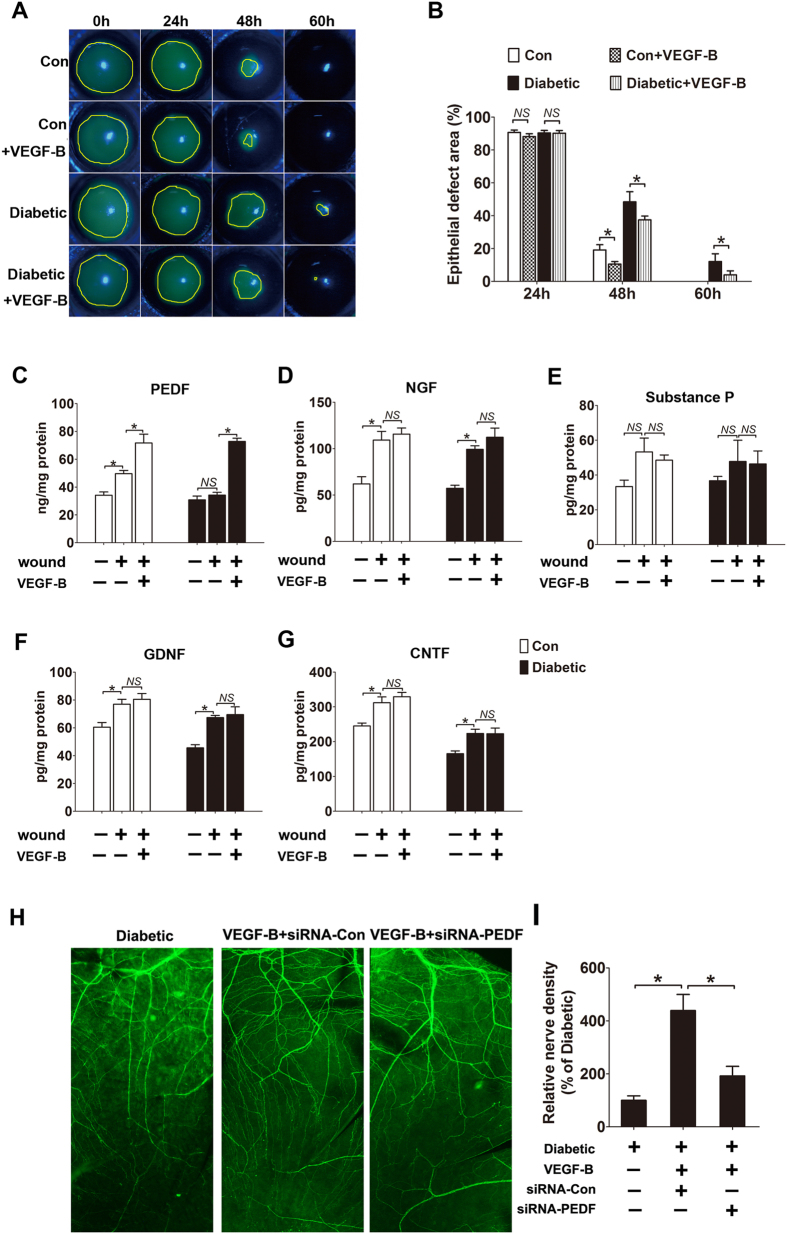
VEGF-B regulates neurotrophic factors release and accelerates corneal epithelial wound healing. Diabetic and control mice were received 2 mm central corneal epithelial debridement as described above, treated with VEGF-B or PBS. (**A,B**) The healing process was visualized with fluorescein staining of the denuded area and analyzed with Image J software (n = 8 per group). (**C–G**) 3 days post injury, corneas were collected and analyzed by indirect ELISA for levels of PEDF, NGF, GDNF, CNTF and substance P (n = 8 per group). (**H**) Diabetic mellitus corneas pretreated with PBS, control siRNA, and PEDF siRNA. Whole corneas of each condition were harvested at indicated time point and stained for βIII-tubulin (n = 4 per group). (**I**) Quantification of corneal nerve regeneration for each group. Data are representative of means ± SD. *p < 0.05.

In diabetic cornea, hyperglycemia-induced corneal innervations dysfunction and neurotrophic deficits were believed to be an important cause of impaired corneal epithelial wound healing^10^,^22^,^23^. To explore the effect and mechanism of VEGF-B on diabetic corneal epithelial wound healing, central corneal epithelium was debrided in diabetic mice and age-matched normal mice with or without VEGF-B application. The rate of corneal re-epithelialization revealed a significant difference from 48 h after corneal epithelial debridement (Fig. 6A). The corneal epithelial defect of VEGF-B-treated diabetic mice (48 h: 39.9 ± 6.9%, 60 h: 5.0 ± 3.9%) was significantly improved from that of vehicle-treated diabetic mice (48 h: 48.4 ± 12.4%, 60 h: 12.6 ± 6.0%) (Fig. 6B). Similarly, VEGF-B injection also promoted the corneal epithelial wound healing in normal mice (Fig. 6A,B). Furthermore, the ELISA results showed that the contents of neurotrophic factor PEDF, NGF, GDNF and CNTF were significantly increased at 72 h after the debridement of corneal epithelium in both normal and diabetic cornea. However, VEGF-B treatment induced a further increasing of corneal PEDF level, but not NGF, GDNF, CNTF or neuropeptide substance P in both normal and diabetic mice (Fig. 6C–G). Moreover, in diabetic cornea, PEDF knock down by siRNA partially, but significantly abolished the neurotrophic function of VEGF-B on corneal reinnervation (Fig. 6H,I), which suggests that PEDF may be the potential trophic factor induced by VEGF-B for the promotion of corneal epithelial wound healing.

## Discussion

The prevalence of diabetic peripheral neuropathy (DPN) highlights the need to develop novel agents capable of stimulating nerve regeneration. Accumulating evidence indicates that the members of vascular endothelial growth factor (VEGF) family provide attractive opportunities for treatment of DPN^24^,^25^,^26^. In the present study, by using a STZ- induced diabetic mouse model, we demonstrate that hyperglycemia reduces the endogenous VEGF-B expression in regenerated corneal epithelium, while exogenous VEGF-B application promoted diabetic corneal nerve regeneration *in vivo* and *in vitro*. Mechanically, VEGF-B reactivated PI-3K/AKT-GSK3β-mTOR signaling and attenuated the oxidative stress in diabetic trigeminal ganglion neurons, mainly through VEGFR-1. Moreover, VEGF-B improved corneal sensation and epithelial regeneration, accompanied with the elevated corneal content of PEDF, but not NGF, CNTF, GDNF or substance P. Taken together, hyperglycemia-suppressed endogenous VEGF-B expression in corneal epithelium contributes to the diabetic corneal neuropathy, while exogenous VEGF-B promoted the recovery of corneal innervations and trophic functions in diabetic mice, suggesting VEGF-B may be used as a therapeutic target for diabetic keratopathy.

Recent evidence suggested VEGF-B as a potent neuroregenerative factor for different types of neuron, however, the molecular mechanism remains elusive^27^,^28^,^29^,^30^. VEGF-B selectively binds and signals through VEGFR-1 and co-receptor NRP-1^17^. Previous studies showed that VEGFR-1 deficient mice developed a more severe form of sensory neuron retrograde degeneration in a model of distal neuropathy. Meanwhile, dorsal root ganglion neurons isolated from VEGFR-1 deficient mice failed to response to VEGF-B when subjected to neurotoxic drugs^27^. In parallel, our data revealed that inhibition of VEGFR-1 using a neutralizing antibody completely abolished VEGF-B-mediated neurotrophic effect. In contrast, co-administration of NRP-1 neutralizing antibody resulted only in a partial inhibition, suggesting that the effects of VEGF-B on diabetic sensory neurons were mediated primary through VEGFR-1. Moreover, several intracellular signaling pathways that involved in axonal survival and regeneration, including the PI-3K/AKT, ERK and mTOR, were impaired in chronic hyperglycemia^31^,^32^,^33^,^34^. Our data revealed that VEGF-B treatment selectively activated PI-3K/AKT, but not ERK, in primary culture of TG neurons. Notably, blocking PI-3K, but not ERK, completely abolished VEGF-B mediated neurite outgrowth, although the phosphorylation of ERK was down-regulated by hyperglycemia. Further studies are needed to address whether the reduced activation of ERK in cultured diabetic trigeminal ganglion neurons is associated with diabetic corneal neuropathy. In addition, pharmacological blockade of GSK3β and mTOR also prevented the VEGF-B-stimulation of axonal regeneration, indicating that activation of VEGFR-1 and the consequent triggering of the intracellular signaling pathways driven by PI-3K/AKT are involved in the neurotrophic action of VEGF-B.

Oxidative stress has been proposed as a primary pathogenic factor responsible for the development and progression of DPN^35^,^36^. Mitochondrial dysfunction induced by chronic hyperglycemia contributes to axonal regenerative failure in diabetic peripheral neuropathy^37^,^38^,^39^. In the current study, we confirmed that the mitochondrial superoxide level was up-regulated in diabetic TG neurons, as well as the loss of mitochondrial membrane potential. Intriguingly, we showed that VEGF-B could mitigate ROS overproduction in sensory neurons of diabetic mice, suggesting that the neuroprotective role of VEGF-B may involve in activating anti-oxidative defense in diabetic sensory neurons. In line with our results, previous studies provided evidence for VEGF-B conferring neuroprotection via free radical scavenging^28^. Moreover, *in vitro* study also showed that VEGF-B treatment could ameliorated ROS generation in cardiomyocytes in response to both angiotensin II and norepinephrine^40^.

The cornea is the most densely innervated structure in the human body and is dominantly supplied by sensory nerve fibers from trigeminal ganglion neurons. Corneal nerve fibers release diffusible trophic factors that maintain the homeostasis of corneal epithelium and stimulate corneal epithelial cell proliferation, migration, differentiation and stratification once injured^41^,^42^,^43^. As previous report, the promotion of VEGF-B on corneal epithelial wound healing was independent on the direct effects on corneal epithelial cells, which the authors suppose to be the accelerated recovery of trophic influence of regenerated corneal nerves by VEGF-B^19^. In the present study, we further examined the corneal contents of neuropeptide and neurotrophic factors that have been reported to be important in corneal epithelial wound healing. The results showed only PEDF, not SP, NGF, CNTF, GDNF, was up-regulated with the treatment of VEGF-B in both normal and diabetic cornea. Consistently, PEDF and its fragment (44-mer PEDF) have been found to accelerate corneal epithelial regeneration by promoting the self-renewal of corneal epithelial stem cells^44^,^45^. Moreover, when combined with docosahexaenoic acids (DHA), PEDF treatment promoted corneal nerve fiber regeneration and improved corneal sensitivity after corneal surgery in animal models^46^,^47^,^48^. The results suggest PEDF may be the potential trophic factor induced by VEGF-B for the promotion of corneal epithelial wound healing, nerve fiber regeneration and sensation improvement in normal and diabetic mice. However, although PEDF blockade by siRNA partially abolished trophic function of VEGF-B in diabetic corneal re-innervation, further studies are needed to clarify whether PEDF is critical or only involved in the improvement of VEGF-B in corneal neuropathy.

In summary, our data demonstrated that endogenous VEGF-B expression was reduced in the regenerated corneal epithelium of diabetic mellitus. Exogenous VEGF-B promoted the diabetic corneal nerve fiber regeneration, sensation restoration and epithelial wound healing. The mechanism was related with the reactivation of PI-3K/Akt-GSK-3β-mTOR signaling, the improvement of neuronal oxidative stress and the releasing of neurotrophic factor PEDF. Given the neurotrophic and minimal angiogenic activity, VEGF-B provides attractive opportunities for treatment of diabetic corneal and other peripheral neuropathy.

## Methods

### Animals

C57BL/6 mice (6–8 weeks old, male) were purchased from Beijing HFK Bioscience Co., LTD (Beijing, China). The animal care and procedures were conducted according to the Principles of Laboratory Animal Care. The use of animals in this study adhered to the ARVO Statement for the Use of Animals in Ophthalmic and Vision Research, and the animal study was approved by the Shandong Eye Institute Ethics Committee for Animal Experimentation (Approval document No. 2012-6, Qingdao, Shandong, China). Type 1 Diabetes mellitus was induced by intraperitoneal injections of low-dose streptozotocin (STZ, 50 mg/kg, Sigma-Aldrich, St. Louis, MO) for 5 consecutive days. Animals with the HbA1c values higher than 6.5% and blood glucose level higher than 16.7 mmol/L were considered DM and used for experiments, at 16 weeks after streptozotocin injection.

### Corneal sensitivity

Corneal esthesiometry was carried out as previous description by using a Cochet-Bonnet esthesiometer (Luneau Ophtalmologie, Chartres Cedex, France)^10^. The nylon monofilament had a maximal extended length of 60 mm with a diameter of 0.12 mm. The central area of cornea was touched once on each eye, beginning with the full length of nylon filament and shortened by 5 mm until a blink response was elicited. The corneal sensitivity threshold was calculated as the mean value of three longest filament lengths causing positive response.

### Corneal epithelial wound healing

Diabetic mice and age-matched normal mice were anesthetized by an intraperitoneal injection of xylazine (7 mg/kg) and ketamine (70 mg/kg) followed by topical application of 2% xylocaine. The central corneal epithelium (2 mm) was removed with an algerbrush II rust ring remover (Alger Co, Lago Vista, TX) and subsequently applied with ofoxacin eye drops to avoid infection. The defects of corneal epithelium were monitored at 24, 48, and 60 hours by instilling 0.25% fluorescein sodium and photographed under a BQ900 slit lamp (Haag-Streit, Bern, Switzerland). The staining area was analyzed by using Image J software and calculated as the percentage of residual epithelial defect. For VEGF-B treatment, normal or diabetic mice were injected subconjunctivally with 0.5 ng VEGF-B_186_ (5 μl/eye, R&D, Minneapolis, MN) in phosphate buffer saline (PBS) just after the debridement of corneal epithelium, while control mice were injected with PBS vehicle. Corneas were harvested 7 days after wounding for the evaluation of corneal nerve fiber regeneration. siRNA targeting PEDF and control siRNA (20 μmol/L, 5 μl/eye, sc-40948, Santa Cruz Biotechnology.) were injected twice (24 h and 4 hours) before wounding, as described before^49^.

### Corneal whole-mount staining

Cornea whole-mount staining was performed as previously described^50^. In brief, full-thickness corneal flat mounts were fixed for 1 h at room temperature in 4% paraformaldehyde (PFA), incubated at 37 °C in 20 mM EDTA (Sigma-Aldrich) for 30 min and permeabilized in 10% TritonX-100 for 1 h at room temperature. To block nonspecific staining, all samples were incubated with 5% bovine serum albumin (Sigma-Aldrich) in PBS for 1 h. Subsequently, they were incubated with a rhodamine-conjugated mouse anti-β-III tubulin antibody (1:100; R&D) for 24 h at 4 °C. Finally, the flat mounts were examined under an Eclipse TE2000-U microscope (Nikon, Tokyo, Japan).

### Corneal nerve density cal**c**ulation

Using a modified version of a method from previous studies^19^,^23^, we examined the density of corneal nerve fibers by using images taken from whole-mount corneas. The central and peripheral cornea nerve densities were calculated and expressed as the total length per unit area (mm/mm^2^) in the observed fields. Following conversion to 8-bit gray scale images, The length of each fiber consisting of the subbasal nerve plexus was measured by tracing it using Neuron J software (a plug-in for Image J), and the total length of the subbasal nerve plexuses in the observed field was presented (see Supplementary Fig. S1). Five image fields were sampled in each corneal whole mount: one at the center cornea and the rest at midway between the central and limbal cornea for each of four corneal quadrants.

### Trigeminal ganglion neuron culture and treatment

Trigeminal ganglion (TG) neurons were isolated and dissociated from TG tissue of diabetic or normal mice as described previously^51^. In brief, ophthalmic branches of the trigeminal ganglion were harvested from diabetic mice and normal control. The tissues were subjected to enzymatic digestion with papain and collagenase/dispase. Trigeminal neurons were separated in Percoll gradients by centrifugation at 2000 rpm for 30 min. The isolated cells were plated onto Poly-D-lysine & laminin-coated dishes and incubated in the neurobasal-A medium (with no D-glucose, Life Technologies, USA) with B27 supplement (Life Technologies). In all *in vitro* assays, the neurons from control mice were grown in the medium supplemented with 10 mM D-glucose (Sigma-Aldrich), while the neurons from diabetic mice were maintained in the medium containing 25 mM D-glucose to mimic the hyperglycemia conditions^37^,^52^. Trigeminal ganglion neurons were treated with 50 ng/ml VEGF-B_186_ for evaluate its effects on neurite outgrowth. For inhibition studies, TG neuron cells were incubated with VEGF receptor 1-neutralizing antibody (2.6 nM, R&D), or NRP-1 neutralizing antibody (2.6 nM, R&D), or PI-3K inhibitor LY294002 (1 μM, Sellect.cn), or ERK inhibitor U0126 (1 μM, Sigma-Aldrich), or GSK3β inhibitor TDZD-8 (1 μM, Sigma-Aldrich), or mTOR inhibitor rapamycin (100 nM, Sigma-Aldrich) for 48 h in the presence of VEGF-B^16^,^19^. For Western blots studies, neurons were cultured for 6 h in the basal medium without supplements, pretreated for 1 h with blocking antibodies or inhibitors and then treated with VEGF-B. Cell lysates were prepared 30 min after VEGF-B addition and analyzed by immunoblotting. All the treatment concentrations used in this study have been evaluated and optimized in our primary experiments.

### Immunohistochemical staining

To visualize the neurite outgrowth, neurons were stained overnight at 4 °C with mouse anti-β-III tubulin antibody (1:500; R&D) and then incubated with Alexa Fluor 488-conjugated IgG (Life Technologies) for 1 h. The fluorescence staining was captured using an Eclipse TE2000-U microscope. Neurite outgrowth was analyzed and calculated by using quantitative nerve tracing software Neuron J. For immunohistochemical staining of VEGF-B, eyeballs from diabetic and control mice were fixed in 4% PFA overnight, and subsequently processed for paraffin imbedding using standard procedures. Antigen retrieval was obtained by heating tissue slides in 0.01 M citrate buffer, pH 6.0, at 95 °C for 20 min, and then endogenous peroxidate activity were blocked by 0.6% hydrogen peroxide for 10 min. Nonspecific staining was blocked by 5% normal goat serum for 1 h. Sections were then incubated with goat anti-VEGF-B (1:200; R&D) overnight at 4 °C. After three washes with PBS, they were incubated with biotinylated rabbit anti-goat IgG (1:100) for 1 h, followed by DAB staining for 2 min. Rabbit primary antibody isotype control (R&D) was applied as negative control.

### Intracellular ROS, Mitochondria superoxide and membrane potential staining

For the observation of intracellular ROS generation, cultured TG neuron cells were loaded with 10 μM fluorescence probe 2,7-dichlorodihydrofluorescein diacetate, acetyl ester (DCHF-DA; Molecular Probes, Eugene, OR) for 30 min at 37 °C, and excess dye was then washed out using KRB solution. The staining was detected and captured using an Eclipse TE2000-U microscope. For the observation of mitochondrial superoxide generation, and membrane potential, the cells were preloaded with 5 μM MitoSOX^TM^ red reagent (Beyotime), and 5 μg/ml 5,5′,6,6′-tetrachloro-1,1′,3,3′-tetraethyl-benzimidazole-carbocyanide iodine (JC-1; Beyotime), respectively, for 15 min at 37 °C. The fluorescence was detected and captured using an Eclipse TE2000-U microscope.

### Western blot assay

Total protein was extracted from the lysed samples of mouse TG or cultured neurons in RIPA buffer. Samples (total protein concentration: 40 μg for mouse TG and corneal tissue or10 μg for cultured neurons) were run on 12% SDS-PAGE gels and then transferred to a PVDF membrane (Millipore, Billerica, MA). The blots were blocked with 5% non-fat dry milk for 1 h at room temperature and incubated overnight at 4 °C with primary antibodies, including VEGF-B (1:500; R&D),VEGFR-1 (1:1000; Abcam), NRP-1 (1:1000; Abcam), p-Akt (1:2000; Abcam), Akt (1:5000; Abcam), p-ERK (1:1000; CST), ERK (1:1000; CST) p-GSK3β (1:2000; CST), GSK3β (1:2000; CST), p-mTOR(1:1000, CST), mTOR(1:1000, CST), p-S6K (1:2000; CST), S6K (1:2000; CST) and GAPDH (1:3000; Abcam). Finally, the blots were incubated with the horseradish peroxidase-conjugated secondary antibody (1:3000; Amersham Biosciences, Piscataway, NJ) and visualized via the enzyme-linked chemiluminescence using the ECL kit (Chemicon, Temecula, CA).

### ELISA assay

Diabetic mice and age-matched normal mice were subjected to 2 mm central corneal epithelial debridement and treated with VEGF-B as described above. Three days later, 8 corneas at each time point were homogenated in 350 μl of cold PBS and analyzed by ELISA kits including pigment epithelial-derived factor (PEDF, USCN Life Science Inc. Wuhan, China), nerve growth factor (NGF, Millipore, Bedford, MA), ciliary neurotrophic factor (CNTF, USCN), glial cell-derived neurotrophic factor (GDNF, USCN) and Substance P (SP, Cayman Chemical, Ann Arbor, MI) according to the manufacturer’s procedures.

### Statistical analysis

Data in this study were representative of at least three different experiments and presented as the means ± SD. Statistical analysis was performed using SPSS 17.0 software (SPSS, Chicago, IL, USA) and one-way ANOVA (analysis of variance). A P value of <0.05 was considered significant.

## Additional Information

**How to cite this article**: Di, G. *et al*. VEGF-B promotes recovery of corneal innervations and trophic functions in diabetic mice. *Sci. Rep.* **7**, 40582; doi: 10.1038/srep40582 (2017).

**Publisher's note:** Springer Nature remains neutral with regard to jurisdictional claims in published maps and institutional affiliations.

## Supplementary Material

Supplementary Information
