# Supplementary material for: VEGF-B promotes recovery of corneal innervations and trophic functions in diabetic mice
Source: Sci Rep. 2017 Jan 16;7:40582. doi: 10.1038/srep40582 (PMC5238415; doi:10.1038/srep40582)
Supplement: Supplementary Information [file srep40582-s1.pdf]

VEGF-B promotes recovery of corneal innervations and trophic functions in diabetic mice

Guohu Di#, Xiaowen Zhao#, Xia Qi, Songmei Zhang, Lu Feng, Weiyun Shi, Qingjun Zhou*

State Key Laboratory Cultivation Base, Shandong Provincial Key Laboratory of Ophthalmology, Shandong Eye Institute, Shandong Academy of Medical Sciences, Qingdao, China.

# contributed equally to this study

*Correspondence: **Qingjun Zhou**

Address: Shandong Eye Institute, 5 Yan'erdao Road, Qingdao, 266071, China.

Email: qjzhou2000@hotmail.com

Phone: 86-532-8589-9270

Fax: 86-532-8589-1110

Supplement 1. Quantitative analysis of corneal subbasal innervation. (A) After the corneas were stained with βIII-tubulin antibody, representative images were acquired from central or peripheral cornea. (B) Images were converted to 8-bit gray scale images. (C) The total length of traced nerves was calculated by using Neuron J software.

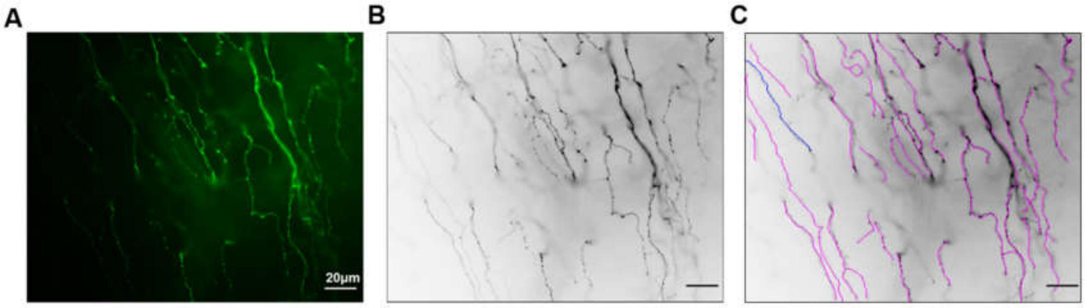

Supplement2. Expression of VEGFR-1 and NRP-1 in trigeminal sensory neurons. TG neurons were isolated and cultured for 4 days. TG neurons were stained with βIII-tubulin antibody, VEGFR-1 antibody and NRP-1 antibody.

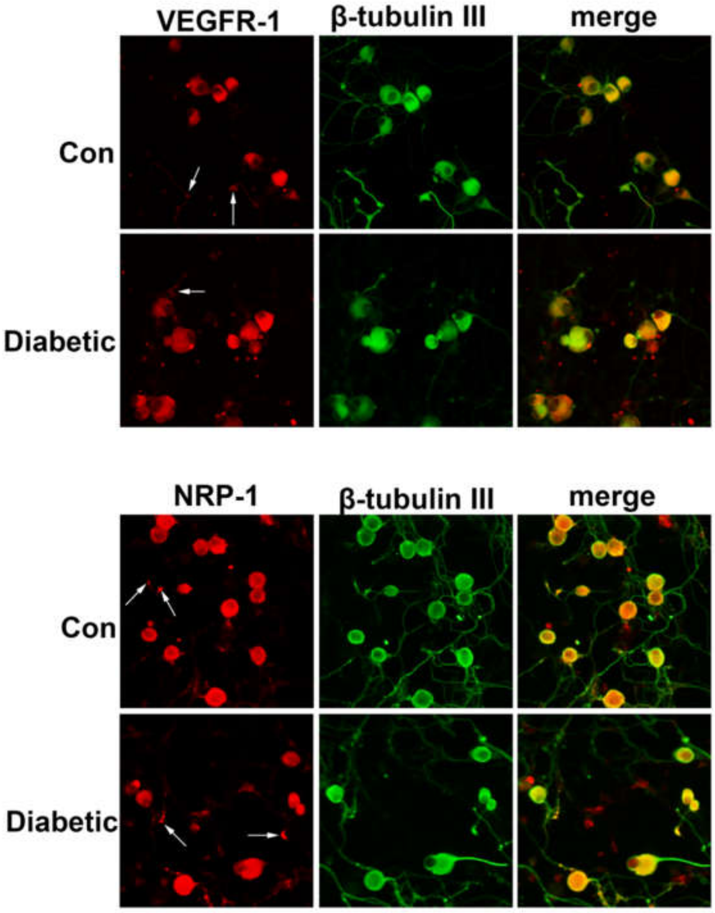

Supplement 3. Quantification of DCF and Mitosox in trigeminal sensory neurons

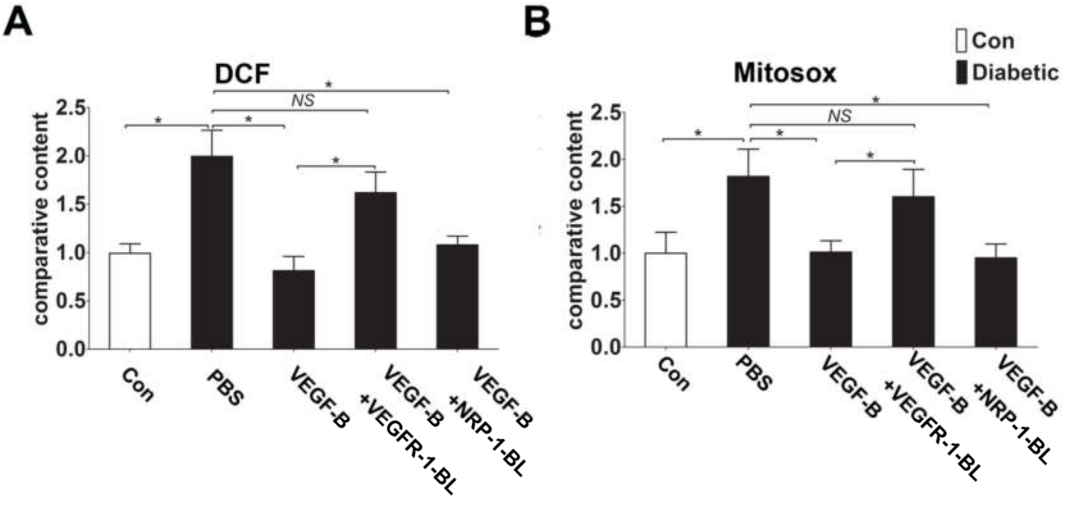
A
DCF
comparative content
2.5
2.0
1.5
1.0
0.5
0.0
*
NS
Con
PBS
VEGF-B
VEGF-B +VEGFR-1-BL
VEGF-B +NRP-1-BL
B
Mitosox
Con
Diabetic
